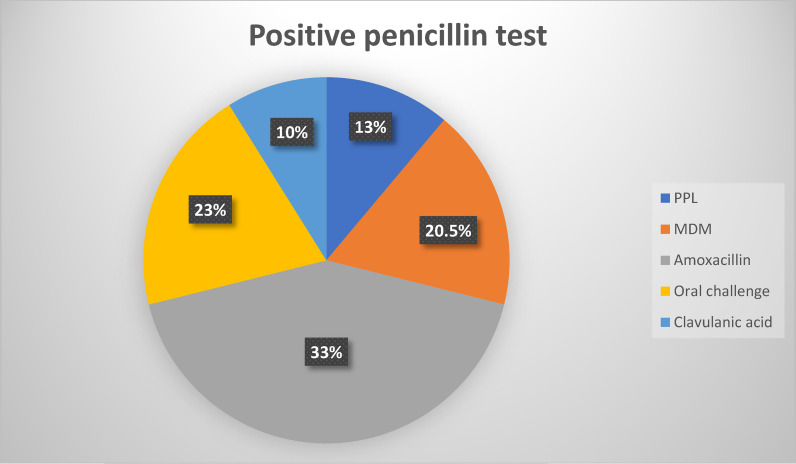# Results of penicillin skin testing in patients with suspected penicillin allergy – the Qatar experience

**DOI:** 10.5339/qmj.2022.fqac.10

**Published:** 2022-04-01

**Authors:** Dalal Mudawi, Salma Taha, Yaldez Ibrahim, Mervat Omar, Hassan Mobayed

**Affiliations:** ^1^Allergy and Immunology Division, Department of Medicine, Hamad Medical Corporation, Doha, Qatar E-mail: DMudawi1@hamad.qa

**Keywords:** amoxicillin/clavulanate, penicillin allergy, penicillin skin testing

## Abstract

Background: Unverified penicillin allergy has been linked to adverse patient events and increased healthcare expenditure owing to the usage of broad-spectrum, expensive antibiotics. Penicillin allergy test is the gold standard to diagnose penicillin allergy; and in this study, we present data from Qatar which have not been published before.

Methods: Patients with a history of penicillin allergy who underwent penicillin allergy testing between January 2015 and December 2020 at the Allergy Division of the Hamad General Hospital were retrospectively reviewed from the division registry. Benzylpenicilloyl-polylysine (PPL) and minor determinant mixture (MDM) kit DAP-penicillin (0.04 mg +0.5 mg)/vial) (penicillin G, amoxicillin (20 mg/vial), and lately clavulanic acid (20 mg/vial) (DAP, Diater, Madrid, Spain) were used for skin and intradermal testing according to published guidelines. Patients with negative skin tests were administered direct oral challenge with amoxicillin/clavulanate (500/125 mg) and observed for 2 hours.

Results: Of the 189 charts reviewed, 183 patients had a complete data set for analysis. Patients were predominantly women (n = 132, 72%) with an average age of 42 years. Of these patients, 149 (81.4%) had a history of an immediate allergic reaction to penicillin, 10 had a history of delayed reactions, and 24 had other or undefined reactions. A total of 39 (21.3%) patients were diagnosed with penicillin allergy (30 patients with positive skin test results and 9 using a direct oral challenge).

Of the 30 patients with positive skin testing, 5 reacted to PPL, 8 to MDM, 13 to amoxicillin, and 4 to clavulanic acid.

Conclusion: Previous studies indicate that 90% patients with a history of penicillin allergy were able to tolerate the drug (10% were truly allergic). Our data showed that 21% were truly allergic to penicillin. This high positive rate can be attributed to the high pretest probability based on the detailed history obtained before the test, which led to the exclusion of patients with symptoms incompatible with penicillin allergy from the test.

## Figures and Tables

**Figure 1.**